# Virome Characterization in Commercial Bovine Serum Batches—A Potentially Needed Testing Strategy for Biological Products

**DOI:** 10.3390/v13122425

**Published:** 2021-12-03

**Authors:** Willian P. Paim, Mayara F. Maggioli, Shollie M. Falkenberg, Akhilesh Ramachandran, Matheus N. Weber, Cláudio W. Canal, Fernando V. Bauermann

**Affiliations:** 1 Department of Veterinary Pathobiology, College of Veterinary Medicine, Oklahoma State University (OSU), Stillwater, OK 74078, USA; ppaimw@gmail.com (W.P.P.); mayara.maggioli@okstate.edu (M.F.M.); 2 Laboratório de Virologia, Faculdade de Veterinária, Universidade Federal do Rio Grande do Sul (UFRGS), Rio Grande do Sul, Porto Alegre 90040-060, Brazil; claudio.canal@ufrgs.br; 3 Ruminant Diseases and Immunology Research, Agricultural Research Service, USA Department of Agriculture, Ames, IA 50010, USA; shollie.falkenberg@usda.gov; 4 Molecular Diagnostic Laboratory, Oklahoma Animal Disease Diagnostic Laboratory, Oklahoma State University (OSU), Stillwater, OK 74078, USA; rakhile@okstate.edu; 5 Laboratório de Microbiologia Molecular, Instituto de Ciências da Saúde, Universidade Feevale, Rio Grande do Sul, Novo Hamburgo 93510-235, Brazil; matheusnweber@gmail.com

**Keywords:** bovine serum, diagnostic, surveillance, viral metagenomics

## Abstract

Bovine serum has been widely used as a universal supplement in culture media and other applications, including the manufacture of biological products and the production of synthetic meat. Currently, commercial bovine serum is tested for possible viral contaminants following regional guidelines. Regulatory agencies’ established tests focused on detecting selected animal origin viruses and are based on virus isolation, immunofluorescence, and hemadsorption assays. However, these tests may fail to detect new or emerging viruses in biological products. High-throughput sequencing is a powerful option since no prior knowledge of the viral targets is required. In the present study, we evaluate the virome of seven commercial batches of bovine serum from Mexico (one batch), New Zealand (two batches), and the United States (four batches) using a specific preparation and enrichment method for pooled samples and sequencing using an Illumina platform. A variety of circular replicase-encoding single-stranded (CRESS) DNA families (*Genomoviridae*, *Circoviridae*, and *Smacoviridae*) was identified. Additionally, CrAssphage, a recently discovered group of bacteriophage correlated with fecal contamination, was identified in 85% of the tested batches. Furthermore, sequences representing viral families with single-stranded DNA (*Parvoviridae*), double-stranded DNA (*Polyomaviridae* and *Adenoviridae*), single-stranded RNA (*Flaviviridae*, *Picornaviridae*, and *Retroviridae*), and double-stranded RNA (*Reoviridae*) were identified. These results support that high-throughput sequencing associated with viral enrichment is a robust tool and should be considered an additional layer of safety when testing pooled biologicals to detect viral contaminants overlooked by the current testing protocols.

## 1. Introduction

Bovine serum has been used as a universal supplement in cell culture applications [1]. The cell culture uses include in-vitro diagnostics, vaccine production, gene therapy, stem cell therapy, and, more recently, the production of synthetic meat [2,3,4]. Different categories of bovine serum are commercially available, including Fetal Bovine Serum (FBS), Newborn Calf Serum (NCS), Bovine Calf Serum (BCS), and Donor Bovine Serum (DBS).

Bovine origin products used to manufacture biologicals are currently tested for viral contaminants following regional guidelines. For instance, in the United States (USA), the test for adventitious viruses in cattle serum is performed in compliance with the United States Department of Agriculture (USDA) regulations for biologics products as specified in the Code of Federal Regulations (CFR9), Title 9, Part 113.53. The USDA regulations require the testing of bovine serum for Bovine Adenovirus (BAV), Bovine Herpesvirus (BoHV), Bovine Parvovirus (BPV), Bovine Respiratory Syncytial Virus (BRSV), Bovine Viral Diarrhea Virus (BVDV), Bluetongue Virus (BTV), Parainfluenza Virus 3 (PI3), Rabies Virus (RABV), and Vesicular Stomatitis Virus (VSV). The screening tests are mainly conducted using traditional virology assays, including virus isolation, immunofluorescence, and hemadsorption. Similarly, in Europe, the use of bovine serum for the manufacture of human medicinal products with a biological origin is regulated by the European Medicines Agency (EMA/CHMP/BWP/457920/2012 Rev.1). The veterinary products in Europe are regulated by the EMA-CVMP-743-00-Rev.2. 

The EMA guidelines mostly rely on virus isolation in cell culture. Additionally, manufacturers are encouraged to test for specific viruses using nucleic acid detection assays and investigate possible emerging viruses. However, specific guidelines and requirements toward this end are unclear. The lack of a systematic and global strategy to unbiasedly monitor for emerging viral threats creates opportunities for viral entry and dissemination within and among countries, with grave implications to animal and human health.

There is an extensive international trade of bovine serum [5]. While part of the serum batches is sterilized by gamma irradiation, the procedure is not required. Commercialized bovine serum is typically pooled serum, frequently composed of hundreds to thousands of individual samples. In the final product label, the described product origin is based on the country where processing and packaging occurred (World Trade Organization–Rules of Origin, Article 3 {b}). The country processing the serum may not necessarily correspond to where the serum was first collected, or the origin of the animals used to collect the serum. 

Sites collecting and processing bovine serum for commercialization may also handle products of various animal species, allowing the chance for contamination among products with viruses from unrelated animal species. This is especially concerning regarding emerging or high-impact animal disease dissemination. Another relevant concern is the presence of human and zoonotic viruses contaminating bovine serum. During collection and processing, humans are involved in the process, and the testing requirements do not consider the accidental or intentional risk for contaminating bovine serum batches with human viruses. 

Second-generation sequencing technologies, including sequencing by synthesis and pyrosequencing platforms, provide the ability to detect known and unknown viruses in pooled samples [6,7,8,9]. These technologies associated with the enrichment of viral nucleic acid potentially increase the sequencing sensitivity. For instance, an evaluation of the virome in FBS samples used the Klenow enzyme for DNA amplification and characterized sequences originating from eukaryotic viruses [6]. On the other hand, multiple displacement amplification (MDA) was used to identify specific viruses and evaluate the test sensitivity in biological samples [10,11]. In the present study, we characterized the viral population in non-gamma irradiated bovine serum batches from Mexico, the USA, and New Zealand. The serum batches were commercially available in the USA and were subjected to a combination of steps previously described to enrich viral nucleic acid in the samples before sequencing [6,7,9,10,11,12]. Results demonstrated the presence of sequences related to several eukaryotic viruses from a wide range of animal species and a great diversity of phages, including members of the recently discovered group of cross-assembly phage (CrAssphage).

## 2. Materials and Methods

### 2.1. Samples of the Commercial Bovine Serum Batches

Seven batches of non-irradiated commercial bovine serum were purchased from three different manufacturers. One FBS from Mexico (FBS1 Mex), two NCS batches from New Zealand (NCS2 NZ and NCS3 NZ), and four batches from the United States, including one FBS (FBS2 USA), two NCS (NCS1 USA and NCS4 USA), and one BCS (BCS1 USA). The batches of commercial bovine serum were stored at −80 °C until processing. In addition, a non-related sample composed of peripheral blood mononuclear cells (PBMC) from 15 two-week-old calves was added. The sample collection was approved by the Institutional Animal Care and Use Committee (IACUC) protocol number IACUC-20-14-STW.

### 2.2. Sample Preparation and Sequencing

A total of 50 mL of each commercial batch of bovine serum was centrifuged at 2000× *g* for 30 min at 4 °C. Prior to centrifugation, the calf PBMC pooled sample was submitted to a freeze and thaw cycle and then diluted to 50 mL in PBS. The supernatants were filtered using a 0.45 μm pore size membrane (Millipore, Darmstadt, Germany). Subsequently, samples were ultracentrifuged on a 25% sucrose cushion at 100,000× *g* for 3 h at 4 °C in a Sorvall AH629 rotor (Thermo Fisher Scientific, Waltham, MA, USA). The pellet containing the viral particles was incubated for 1.5 h with DNase and RNase enzymes (Thermo Fisher Scientific) as previously described [13]. Viral RNA was isolated using TRIzol™ LS Reagent (Thermo Fisher Scientific), whereas the DNA extraction employed a phenol-chloroform protocol [14]. 

The viral DNA was enriched through multiple displacement amplification (MDA), using a GenomePlex^®^ Complete Whole Genome Amplification (WGA) Kit (Sigma-Aldrich, St. Louis, MO, USA). Following the manufacturer’s recommendations, the viral RNA was reverse-transcribed and further enriched using the SeqPlex RNA Amplification Kit (Sigma-Aldrich). The enriched DNA products were pooled in equimolar amounts and purified using the PureLink™ Quick Gel Extraction and PCR Purification Combo Kit (Thermo Fisher Scientific). The quality and quantity of the DNA were assessed through spectrophotometry and fluorometry performed with NanoDrop™ (Thermo Fisher Scientific) and Qubit™ 4 (Thermo Fisher Scientific). Using the Nextera XT DNA Library Preparation Kit, DNA libraries were prepared with 1 ng of purified DNA. Sequencing was conducted using the Illumina iSeq 100 System using the Illumina i1 reagent kit (2 × 150 paired-end reads).

### 2.3. Bioinformatic Analysis

The quality of the sequences generated was evaluated using the FastQC tool. High-quality sequences (Phred score ≥ Q30) were trimmed on the Illumina iSeq 100 System at the time the FASTQ was generated. The data was de novo assembled on BaseSpace Cloud (Illumina) with the metaSPAdes genome assembler (Version 3.0). All assemblies were confirmed by mapping reads to contigs as previously described [7] and using Geneious Prime software (Version 2020.2.1). The assembled contigs were examined in search of similarities to known sequences with BLASTN software using MegaBLAST [15]. Sequences with *E*-values of ≤10^−5^ were classified as likely originating from a eukaryotic virus, bacteria, phages, eukaryote, or unknown, based on the taxonomic origin of the sequence with the best *E*-value as previously described [16]. Open reading frames (ORF) predictions and genome annotations of the near-full-length genomes were performed using Geneious Prime software. The alignment of contigs and reads to a reference genome was used to generate the near near-full-length genomes using Geneious Prime software (Version 2020.2.1). Gene comparisons were performed with BLASTN (https://blast.ncbi.nlm.nih.gov/Blast.cgi, accessed on 25 January 2021). Representative sequences of viruses belonging to the families *Parvoviridae* and *Polyomaviridae* were obtained from GenBank and aligned with the sequences identified in the present study using MUSCLE software [17]. Phylogenetic trees were constructed using MEGA6 [18], applying the Neighbor-Joining (NJ) method through the p-distance model for *Parvoviridae* analysis and the Maximum-Likelihood (ML) method, General Time Reversible (GTR) model to *Polyomaviridae* family. All analyses were conducted with 1000 bootstrap replicates. 

## 3. Results

### 3.1. Overview

**Figure 1:**
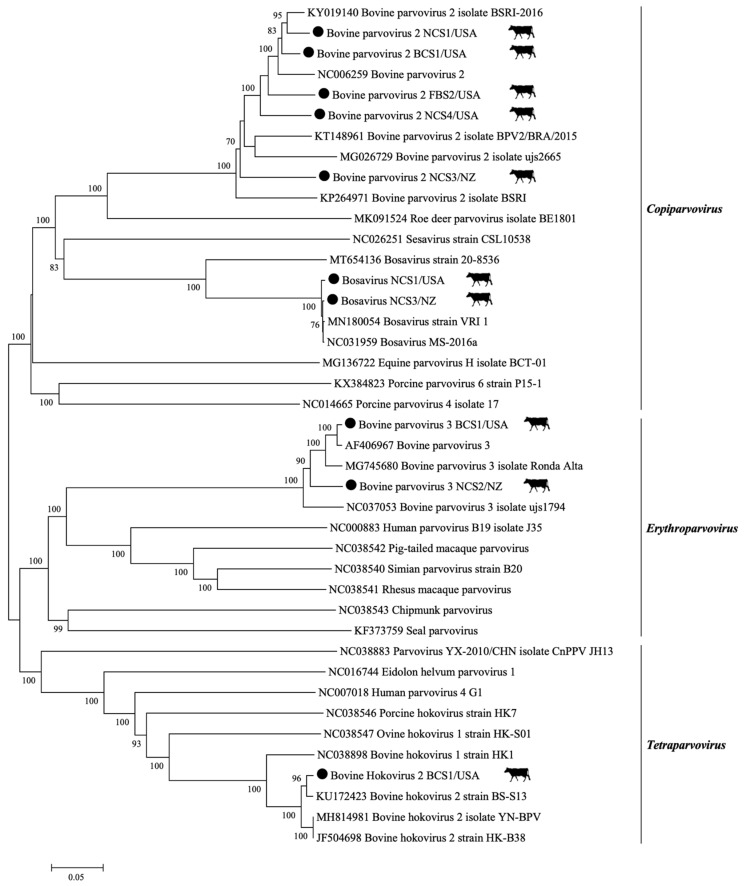
Genetic characterization of members of the *Parvoviridae* family was detected in the present study. Representative genomes belonging to the *Parvoviridae* family were selected from GenBank at the National Center for Biotechnology Information (NCBI). The nucleotide phylogenetic tree was constructed using the Neighbor-Joining (NJ) method based on a p-distance model using the full coding genome. Analyses were conducted with 1000 bootstrap replicates. Complete coding genomes obtained are indicated by the black dots and the sequences were deposited in GenBank under the accession numbers MZ502227, MZ502228, MZ502229, MZ502230, MZ502231, MZ502232, MZ502233, MZ502234, MZ502235, and MZ502236.

**Figure 2:**
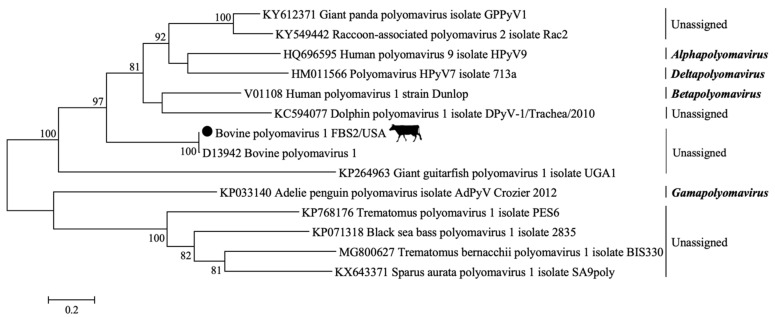
Phylogenetic tree based on the coding sequence of the polyomavirus large tumor antigen gene. The MEGA6 app suite was used for the phylogeny inference using the Maximum-Likelihood (ML) method based on General Time Reversible (GTR) model. Analyses were conducted with 1000 bootstrap replicates. The polyomavirus reference sequences described in the International Committee on Taxonomy of Viruses were retrieved from the GenBank database. The retrieved bovine polyomavirus 1 (BPyV-1) sequence is represented by the black dot and it was deposited in GenBank under the accession number MZ520135.

**Figure 3:**
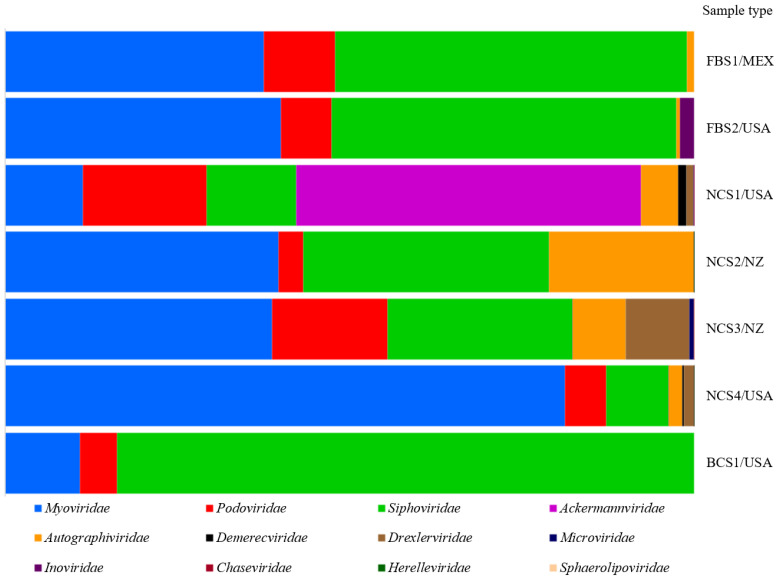
Taxonomic classification of bacteriophage reads. Bar chart representation of the reads based on BLASTN, *E*-value < 1 × 10^−5^ against the GenBank non-redundant database. Sample type: fetal bovine serum (FBS), newborn calf serum (NCS), and bovine calf serum (BCS). Serum origin: Mexico (Mex), New Zealand (NZ), and the United States of America (USA).

Approximately 11,492,156 sequence reads were generated by metagenomic sequencing in the serum batches. From the total retrieved reads, about 12.7% were classified as viral sequences, whereas about 56.8% matched the bovine genome. After assembly, a total of 639,902 contig sequences were obtained; 5.9% were viral contigs. From these, 42.9% were assigned to eukaryotic viruses and 57.1% to bacteriophages. A taxonomic classification, including the number of reads recovered for each eukaryotic virus-related contig, is provided in Table 1. Sequences representing viral families with single-stranded DNA (ssDNA) (*Parvoviridae*), double-stranded DNA (dsDNA) (*Polyomaviridae* and *Adenoviridae*), and circular Rep-encoding single-stranded (CRESS) DNA (*Genomoviridae, Circoviridae*, and *Smacoviridae*) were observed. Additionally, sequences belonging to four RNA viral families were identified, including single-stranded RNA (ssRNA) (*Flaviviridae*, *Picornaviridae*, and *Retroviridae*) and double-stranded RNA (dsRNA) (*Reoviridae*) (Table 1). The complete coding genomes were obtained for Bovine Parvovirus 2 (BPV-2), Bovine Parvovirus 3 (BPV-3), Bosavirus (BosaV), Bovine Hokovirus 2 (BHoV-2) (Figure 1), and Bovine Polyomavirus 1 (BPyV-1) (Figure 2). Most of the viral sequences obtained shared a high degree of identity with known animal viruses. Moreover, sequences representing 12 viral families of bacteriophages were identified (Figure 3), including sequences related to CrAssphage (Table 2). The sequencing results of the PBMC from calves revealed the presence of eukaryotic virus-related contigs belonging to the families *Parvoviridae*, *Herpesviridae*, *Retroviridae*, *Phycodnaviridae*, *Polydnaviridae*, and *Poxviridae.* Additionally, bacteriophage related-sequences from the families *Myoviridae*, *Podoviridae*, and *Siphoviridae* were retrieved (Supplementary Table S1).

### 3.2. DNA Viral Families: Parvoviridae, Polyomaviridae, and Adenoviridae

A high diversity of sequences matching members of *Parvoviridae* were retrieved, including sequences representing four parvovirus genera: *Erythroparvovirus*, *Copiparvovirus, Tetraparvovirus*, and *Bocaparvovirus*. A total of 16,156 contig sequences closely related to the family *Parvoviridae* were observed in the tested samples (Table 1). Among these contig sequences, 11,287 were related to BPV-3 (*Ungulate erythroparvovirus 1*). Two complete coding genomes of BPV-3 were recovered by mapping the contigs and reads to a reference sequence. The BPV-3 genomes were 5100 nucleotides in length, with 58,148× and 10,207× average coverage. The genomes BPV-3 NCS2/NZ and BPV-3 BCS1/USA were deposited in GenBank under the accession numbers MZ502232 and MZ502233, respectively. The *Parvoviridae* family phylogeny is presented in Figure 1.

A total of 4342 contigs were closely related to BPV-2 (*Ungulate copiparvovirus 1*) (Table 1). Five complete coding genomes belonging to the *Copiparvovirus* genus were obtained (BPV-2 FBS2/USA, BPV-2 NCS1/USA, BPV-2 NCS3/NZ, BPV-2 NCS4/USA, and BPV-2 BCS1/USA). The sequences were deposited in GenBank under the accession numbers MZ502227, MZ502228, MZ502229, MZ502230, and MZ502231. The genome lengths were 5334, 5613, 5559, 5257, and 5613 nucleotides, and presented an average coverage of 94 ×, 38,275×, 2150×, 23×, and 10,394×, respectively. Sequences of BosaV (*Ungulate copiparvovirus 5*) were also described in all NCS samples. Two complete coding genomes of *Copiparvovirus* were retrieved (BosaV NCS1/USA and BosaV NCS3/NZ). The genomes were deposited in GenBank under the accession numbers MZ502234 and MZ502235. The genomes were 5222 and 5371 nucleotides long and had an average coverage of 105× and 541×. 

Three serum batches (NCS1/USA, NCS4/USA, and BCS1/USA) showed 106 contig sequences related to bovine hokovirus (Table 1). Notably, nucleotide BLAST demonstrated that 12 contigs presented 87.8% to 100% identity with the Bovine Hokovirus 1 (BHoV-1) strain HK1 and Brazilian BHoV-1 isolate Guarapuava (GenBank accession numbers NC038898 and MG745679), while 94 contigs were related to the Chinese Bovine Hokovirus 2 (BHoV-2) strain HK-B38, and BHoV-2 strain BS-S13 (85.7–100% identity) (GenBank accession numbers JF504698 and KU172423) (Table 1). One complete *Tetraparvovirus* coding genome with 5097 nucleotides in length and 376× average coverage sequence was identified in the BCS1 sample. The BHoV-2 BCS1/USA genome sequence was deposited in GenBank under the accession number MZ502236.

Twenty-six contig sequences that were closely related to the *Polyomaviridae* family were retrieved from serum batches FBS1, FBS2, NCS3, and NCS4. One complete coding genome of BPyV-1 with 19× average coverage of the genome and 4697 nucleotides long was retrieved from FBS2/USA (GenBank accession number MZ520135). The phylogeny is shown in Figure 2.

Four contig sequences were closely related to the *Adenoviridae* family (Table 1). The batch NCS3/NZ showed two contig sequences that were 103 and 147 nucleotides long and 97.1–99.3% nucleotide identity with the Bovine Adenovirus 4 (BAdV-4) strain THT/62 identified in Hungary (GenBank accession number AF036092). The other two contig sequences were detected in serum batch BCS1/USA. The contig sequences were 280 and 282 nucleotides long and were related to the Bovine Adenovirus 6 (BAdV-6) strain 671130 from the Netherlands, with 98.9–97.8% nucleotide identity (GenBank accession number JQ345700).

### 3.3. RNA Viruses: Flaviviridae, Picornaviridae, Reoviridae, and Retroviridae

Four contig sequences were closely related to members of the *Flaviviridae* family, the *Pestivirus A* and *B* species (formerly classified as *Bovine Viral Diarrhea Virus 1* (BVDV-1) and *2* (BVDV-2) species) (Table 1). The serum batches NCS1 and NCS4 contained three contig sequences related to the Bovine Kobuvirus (BKV; species *Aichivirus B*, genus *Kobuvirus*, family *Picornaviridae*) (Table 1). 

A total of three contig sequences closely related to family *Reoviridae* were observed in serum batches NCS2/NZ (two contigs) and NCS3/NZ (one contig). From these, a total of two contig sequences were related to segments 1 and 8 (VP1 and NSP2) of the Bovine Rotavirus A (BRV-A), while one contig (NCS3/NZ) showed 96.1% nucleotide identity to segment 3 (VP3) of the Human Rotavirus (HRV) strain RVA/Human-wt/AUS/V585/2011/G10P (GenBank accession number JX567762). In addition, one contig sequence closely related to the *Retroviridae* family was observed in serum batch FBS1 from Mexico. The 206 nucleotides long contig had 99.5% nucleotide identity with the gag-pol polyprotein from the Moloney Murine Leukemia Virus (MoMLV) (GenBank accession number NC001501).

### 3.4. Circular Rep-Encoding Single-Stranded (CRESS) DNA Viruses-Genomoviridae, Circoviridae, and Smacoviridae

A total of 28 contig sequences closely related to CRESS-DNA viruses were retrieved. Fifteen contig sequences were related to ten CRESS-DNA viruses (feces associated gemycircularvirus 14 and 16, Gopherus associated genomovirus 1, Mongoose feces-associated gemycircularvirus A and B, Sewage-associated gemycircularvirus 3 and *Genomoviridae* sp.) belonging to the *Genomoviridae* family (Table 1). Twelve contig sequences (103–482 nucleotides long) were related to nine CRESS-DNA viruses in the *Circoviridae* family (Cyclovirus Equ1, Po-Circo-like virus 21, Po-Circo-like virus 22, Po-Circo-like virus 41, Po-Circo-like virus S20, Po-Circo-like virus GX19, Starling circovirus, Bo-Circo-like virus CH, and *Circovirus* sp. isolate PoCirV VIRES YN02 C2). The *Smacoviridae* family showed one contig sequence that was 151 nucleotides in length and shared 88.7% nucleotide identity to porcine associated porprismacovirus 10 isolate 49-Fec25-pig detected in NZ (GenBank accession number NC030126). 

### 3.5. Bacteriophages

Viruses from the *Myoviridae*, *Siphoviridae*, and *Podoviridae* families represented the majority of the sequences recovered (Figure 3). A total of 21,566 contig sequences were assembled and closely related to bacteriophages. The *Podoviridae* family had 3361 related contig sequences. Among these contigs, 242 were closely related to CrAssphage and found in six out of the seven tested batches. The NCS serum batches demonstrated an increased number of CrAssphage sequences (Table 2).

## 4. Discussion

Commercial bovine serum is widely used as a supplement for cell culture in research, biotechnology, pharmaceutical manufacturing, and the production of synthetic meat [1,2,3,4]. The extensive international trade of bovine serum and other biologicals poses a possible risk of transporting and disseminating viruses into new regions. Limited consideration is given to possible emerging viruses in the existing safety guidelines, and the detection methodology currently in place is biased toward known targets. Furthermore, the pooled nature of most biological products poses an additional limitation for pathogen identification due to the dilution factor. To overcome these challenges, the present study used metagenomics associated with a combination of viral enrichment steps to comprehensively characterize the virome of seven non-irradiated commercial bovine serum batches from Mexico, New Zealand, and the USA. 

The significant diversity of the virome identified and the retrieval of several near-full length genomes demonstrated the approach’s robustness. Sequences related to vertebrate viruses from families *Parvoviridae*, *Polyomaviridae*, *Adenoviridae*, *Flaviviridae*, *Picornaviridae*, *Reoviridae*, and *Retroviridae* were identified. Additionally, CRESS-DNA viruses (*Genomoviridae*, *Circoviridae*, and *Smacoviridae*) and sequences related to the recently described CrAssphage were recognized. Although it is not possible to confirm the absence of sample contamination during processing in the laboratory, the analyses of the unrelated sample (PBMC) suggest that if contamination occurred, it was limited based on the minimal overlap of viral species identified (Supplementary Table S1).

Due to the small size, viruses from the *Parvoviridae*, and *Polyomaviridae* families, and the CRESS-DNA viruses are less susceptible to the triple 0.1 µm filtration steps usually employed for serum sterilization. As an additional safety precaution, many institutions employ the use of gamma-irradiated serum. The gamma irradiation effect on virus inactivation [19] is variable depending on the virus structure. Studies have demonstrated that the typical dose of irradiation, 25–40 kGy (2.5–4.0 Mrad), may only lead to a minor viral titer reduction for parvoviruses, polyomaviruses, and circoviruses; in some cases limited to reducing about 1 or 2 logs of the original tissue culture infectious dose (TCID_50_) [20,21]. Reoviruses and retroviruses also demonstrated resistance to gamma irradiation, with a titer reduction of about 3 to 4 TCID_50_ logs [22,23]. One limitation of metagenomics is the inability to determine whether the viral sequences were from infectious or inactivated viral particles or free nucleic acid. Despite the ultracentrifugation and nucleases treatment, a significant number of reads matching the host genome were identified and demonstrated that free nucleic acids were sequenced.

Previous studies have identified a reduced number of CRESS-DNA virus species in bovine serum [24,25]. In a virome evaluation of pooled cattle plasma, the DNA synthesis was conducted using a Klenow fragment DNA polymerase, and a total of three groups of CRESS-DNA viruses was identified [24]. The lack of specific enrichment may also have limited the identification of CRESS-DNA families in commercial bovine serum [8]. In the current study, the enrichment of viral DNA was accomplished using the MDA method. A total of 17 CRESS-DNA viral species belonging to the *Genomoviridae*, *Circoviridae*, and *Smacoviridae* families were identified. The enrichment of viral DNA using the MDA method may have favored the circular DNA viral species [26], and therefore, the relative abundance of reads identified may not represent the abundance of virus and/or nucleic acid in the original sample. Whereas the tested serum samples may have a variable number of contaminating viral species in each study, the significantly lower number of viral species identified in other studies may suggest that a combination of viral enrichment is essential before sequencing. Moreover, due to the dilution factor of commercial bovine serum samples, ultracentrifugation of larger amounts of the product prior to nucleic acid extraction may also have favored the increased diversity of viral species in the present study compared to direct nucleic acid extraction [11]. 

The quality of bovine serum is also assessed by the endotoxin concentration, which may correlate to the initial bacterial load on the collected samples and translate into the overall sanitary conditions employed during collection and processing [27]. While increased endotoxin levels may indicate poor sanitary conditions, no further insights on the origin of bacterial contamination can be retrieved. Among the bacteriophages identified in the present study, CrAssphage was identified in six out of the seven tested batches. CrAssphage has not been reported as a contaminant of bovine serum in previous studies [8,24,25]. This recently discovered bacteriophage infects *Bacteroides intestinalis* [28,29], and was found to be one of the most abundant human gut phages. In addition to human feces, CrAssphage has been detected in non-human primates and several mammal fecal samples [28,30,31,32,33,34,35,36]. Due to its correlation with fecal material, its presence was suggested as a potential marker for fecal contamination [30,32,37,38,39]. The presence of CrAssphage in the bovine serum lots is likely due to contamination during collection or processing and packing. However, this study cannot determine the precise source of CrAssphage contamination in the tested serum batches.

The ever-growing bovine serum market may lead to product shortage and induce the use of lower quality serum or products with unreliable or untraceable origin. Furthermore, product scarcity associated with the biased testing guidelines and the lack of specific search for viruses from other animal species, including humans, are concerning. Additionally, the current guidelines overlook testing for viruses potentially involved with the disease in humans, like the CRESS DNA viruses. The results demonstrated a high diversity of sequences related to eukaryotic viral families, including CRESS DNA viruses belonging to the *Genomoviridae*, *Circoviridae,* and *Smacoviridae* families. Moreover, representative sequences for 12 bacteriophage families were identified, including related sequences for CrAssphage. Considering the limitations of the current testing, our results indicate that high-throughput sequencing and viral enrichment steps can be a valuable supportive method to attest to the safety and quality of pooled biological products while supporting viral surveillance and early detection of emerging threats.

## Figures and Tables

**Table 1:** Summary of sequences retrieved in the commercial batches of bovine serum that matched with eukaryotic viruses.

Sample Type ^a^	No. of Raw Reads	% of Viral/Non-Viral Reads	Best BLASTN Hit ^b^	Non-Assembled Reads ^c^	Assembled Reads	Contig Length Range (nt)	Contig Identity ^d^	Genome Coverage ^e^
FBS1 MEX	772,754	0.004/99.996	Bovine parvovirus 3	18	4	181–311	93.7%	28.2%
			Bovine polyomavirus 1	2	1	205	99.0%	4.7%
			Moloney murine leukemia virus	12	1	206	99.5%	3.3%
FBS2 USA	1,538,378	0.117/99.882	Bovine parvovirus 2	1138	43	107–1904	97.7%	100%
			Bovine parvovirus 3	3	1	219	89.0%	8.6%
			Bovine polyomavirus 1	660	21	106–2250	100%	99.4%
			Bovine viral diarrhea virus 1	5	2	129–180	93.0%	2.5%
			Bovine viral diarrhea virus 2	6	2	151–224	94.7%	2.4%
NCS1 USA	3,157,462	0.453/99.546	Bovine bocaparvovirus 2	878	16	122–3110	100%	88.2%
			Bosavirus	1806	34	101–3021	100%	100%
			Bovine hokovirus 1	41	4	120–2179	5.0%	46.9%
			Bovine hokovirus 2	39	1	395	98.1%	45.6%
			Bovine kobuvirus	4	2	151–207	95.4%	4.4%
			Bovine parvovirus 1	8	1	420	96.0%	12.8%
			Bovine parvovirus 2	11,470	297	101–2552	100%	100%
			Bovine parvovirus 3	67	13	113–623	97.7%	59.2%
			Cyclovirus Equ1	4	2	103–209	100%	17.5%
			Feces associated gemycircularvirus 14	2	1	192	97.4%	9.4%
			Gopherus associated genomovirus 1	2	1	181	82.3%	10.1%
			Mongoose feces-associated gemycircularvirus b	2	1	181	86.2%	8.2%
			Po-Circo-like virus 21	2	1	246	93.3%	6.2%
			Porcine associated porprismacovirus 10	2	1	151	88.7%	5.7%
NCS2 NZ	2,806,994	35.240/64.759	Bosavirus	18	4	157–411	100%	26.7%
			Bovine parvovirus 2	44	11	281–1951	86.0%	25.3%
			Bovine parvovirus 3	989,123	7700	100–2471	100%	100%
			Bovine rotavirus A	5	2	113–157	97.3%	7.7%
			Starling circovirus	2	1	188	95.2%	9.1%
NCS3 NZ	1,082,206	2.081/97.918	Bovine bocaparvovirus 2	4	1	245	100%	5.0%
			Bosavirus	20,458	365	100–2050	100%	100%
			Bovine adenovirus 4	4	2	103–147	99.3%	0.8%
			Bovine parvovirus 2	2002	232	101–1370	96.6%	100%
			Bovine polyomavirus 1	10	2	141–221	100%	8.6%
			Bovine rotavirus A	4	1	298	95.3%	9.0%
			Feces associated gemycircularvirus 14	10	3	190–238	99.6%	37.7%
			Feces associated gemycircularvirus 16	2	1	140	92.1%	6.3%
			Gopherus associated genomovirus 1	2	1	200	79.0%	9.2%
			Human rotavirus A	4	1	103	96.1%	4.0%
			Mongoose feces-associated gemycircularvirus a	2	1	176	80.7%	8.4%
			Mongoose feces-associated gemycircularvirus b	22	4	387–571	99.2%	88.2%
			Sewage-associated gemycircularvirus 3	2	1	162	98.8%	7.6%
NCS4 USA	1,099,344	0.021/99.978	Bovine hokovirus 1	2	1	233	88.6%	4.6%
			Bovine kobuvirus	2	1	203	94.1%	2.5%
			Bovine parvovirus 2	217	26	149–1679	98.4%	100%
			Bovine polyomavirus	6	2	115–233	97.6%	13.0%
			Bovine serum-associated circular virus	4	1	370	92.0%	73.0%
			Genomoviridae sp. ctca367	2	1	119	96.6%	5.4%
BCS1 USA	1,035,018	41.878/58.121	Bovine adenovirus 6	6	2	280–282	98.9%	2.9%
			Bovine hokovirus 1	34	7	107–184	100%	15.3%
			Bovine hokovirus 2	15,009	93	105–1418	100%	100%
			Bovine parvovirus 2	149,979	3733	100–2888	100%	100%
			Bovine parvovirus 3	268,265	3569	100–2574	100%	100%
			Bo-Circo-like virus CH	6	1	149	100%	3.8%
			Circovirus sp. PoCirV VIRES YN02 C2	3	1	334	91.3%	31.1%
			Po-Circo-like virus 21	12	3	132–252	95.5%	14.4%
			Po-Circo-like virus 22	13	1	192	88.5%	4.9%
			Po-Circo-like virus 41	3	1	191	93.6%	6.5%
			Po-Circo-like virus S20	7	1	262	96.5%	38.9%
			Po-Circo-like virus GX19	11	1	480	90.2%	12.2%

^a^ Sample type: fetal bovine serum (FBS), newborn calf serum (NCS), and bovine calf serum (BCS). Serum origin: Mexico (Mex), New Zealand (NZ), and the United States of America (USA). ^b^ Analysis performed in BLASTN (*E*-value < 1 × 10^−5^). ^c^ Number of read sequences obtained by mapping to the virus RefSeq database (NCBI Reference Sequence Database). ^d^ Highest percentage of nucleotide identity based on the reference strain available in the virus RefSeq database (NCBI Reference Sequence Database). ^e^ Coverage of the genome based on the length of the reference strain included on the virus RefSeq database (NCBI Reference Sequence Database). The analyses included contig and reads.

**Table 2:** Characterization of retrieved CrAssphage contig sequences.

Sample Type ^a^	Number of Contigs	Contig Length Range	Best BLASTN Hit ID of the Longest Contig ^b^/Sample Type/Species	Contig Identity ^c^
FBS1 Mex	0	Not apply	Not apply	Not apply
FBS2 USA	1	80	Uncultured crAssphage -NC_024711/Fecal/human	86.08%
NCS1 USA	69	76-625	CrAssphage FA1-2_000172F-MK415404/Gut/human	99.20%
NCS2 NZ	56	108–409	CrAssphage YS1-2_2437-MK415410/Gut/human	98.78%
NCS3 NZ	107	64–721	CrAssphage LMMB-MT006214/Fecal/human	94.59%
NCS4 USA	8	100–241	CrAssphage sp. C0531BW4-MW067003/Fecal/human	97.93%
BCS1 USA	1	74	CrAssphage YS1-2_2437-MK415410/Gut/human	82.43%

^a^ Sample type: fetal bovine serum (FBS), newborn calf serum (NCS), and bovine calf serum (BCS). Serum origin: Mexico (Mex), New Zealand (NZ), and the United States of America (USA). ^b^ Analysis performed in BLASTN (*E*-value < 1 × 10^−5^). ^c^ Nucleotide identity based on the reference strain available in the virus RefSeq database (NCBI Reference Sequence Database).

## Supplementary Materials

The following are available online at https://www.mdpi.com/article/10.3390/v13122425/s1, Table S1: Summary of sequences retrieved in the control sample (PBMC) that matched with eukaryotic viruses and bacteriophages.
