# Supplementary material for: Virome Characterization in Commercial Bovine Serum Batches—A Potentially Needed Testing Strategy for Biological Products
Source: Viruses. 2021 Dec 3;13(12):2425. doi: 10.3390/v13122425 (PMC8705701; doi:10.3390/v13122425)
Supplement: Supplementary file 1 [file viruses-13-02425-s001.zip › viruses-1468110-supplementary.pdf]

Supplemental Table S1. Summary of sequences retrieved in the control sample (PBMC) that matched with eukaryotic viruses and bacteriophages.

Sample type[a]	Family	Best BLASTN hit[b]	Non-assembly reads[c]	Assembly reads	Contig length range (nt)	Contig identity [d]	Genome coverage[e]
PBMC	*Parvoviridae*	Adeno-associated virus 5	23	3	233-285	99.6%	16.7%
		Desmodus rotundus parvovirus	24	1	288	74.3%	6.7%
	Phycodnaviridae	Aureococcus anophagefferens virus	9	1	142	71.8%	0.04%
		Chrysochromulina ericina virus	8	1	239	72.4%	0.1%
		Dishui lake phycodnavirus 1	7	1	271	69.7%	0.2%
		Emiliania huxleyi virus 86	4	1	246	68.7%	0.06%
	Retroviridae	Baboon endogenous virus	4	1	145	71.3%	1.7%
		Jaagsiekte sheep retrovirus	4	1	167	76.6%	2.2%
		Simian retrovirus 8	7	1	316	67.7%	3.9%
		Porcine endogenous retrovirus E	6	2	249-253	75.1%	6.2%
	Polydnaviridae	Diolcogaster facetosa bracovirus	8	1	176	72.2%	0.6%
		Glypta fumiferanae ichnovirus	8	1	294	68.4%	9.4%
		Glyptapanteles indiensis bracovirus	6	1	119	78.2%	0.3%
	Herpesviridae	Human betaherpesvirus 6A	10	1	157	73.9%	0.1%
	Poxviridae	Orf virus	423	19	104-124	80.6%	0.1%
		Volepox virus strain CA	10	1	257	69.6%	0.1%
	Siphoviridae	Bacteriophage N15	8	1	201	73.6%	0.4%
		Enterobacteria phage lambda	44	2	240-708	100%	0.5%
		Enterobacteria phage phi80	4	1	236	100%	0.5%
		Escherichia phage Lambda ev243	4	1	133	71.4%	0.3%
		Proteus phage VB_PmiS-Isfahan	2172	3	134-546	98.2%	1.6%
		Pseudomonas phage phiPSA1	4	1	197	73.6%	0.4%
		Stx2-converting phage Stx2a	26	2	137-344	99.7%	0.8%
	Myoviridae	Escherichia phage RCS47	6	1	384	68.5%	0.3%
		Klebsiella phage ST147-VIM1phi7.1	2	1	229	76.0%	0.7%
		Vibrio phage KVP40	4	1	203	70.4%	0.08%
	Podoviridae	Salmonella phage SEN22	10	1	320	94.1%	0.8%

[a] Sample type: Control sample (CS). [b]Analysis performed in BLASTN (*E*-value < 1×10^{-5}). [c]Number of read sequences obtained by mapping to the virus RefSeq database (NCBI Reference Sequence Database). [d]Nucleotide identity based on the reference strain available in the virus RefSeq database (NCBI Reference Sequence Database). [e]Coverage of the genome based on the length of the reference strain included on the virus RefSeq database (NCBI Reference Sequence Database). The analyses included contig and reads.
